# Melatonin: Effects on Cartilage Homeostasis and Therapeutic Prospects in Cartilage-related Diseases

**DOI:** 10.14336/AD.2020.0519

**Published:** 2021-02-01

**Authors:** Wen-qing Xie, Song-feng Chen, Xiao-hua Tao, Li-yang Zhang, Pei-wu Hu, Wei-li Pan, Yi-bin Fan, Yu-sheng Li

**Affiliations:** ^1^Department of Orthopedics, Xiangya Hospital, Central South University, Changsha, Hunan 410000, China.; ^2^Department of Orthopedics, The First Affiliated Hospital of Zhengzhou University, Zhengzhou 450000, Henan, China.; ^3^Department of Dermatology, Zhejiang Provincial People’s Hospital, People’s Hospital of Hangzhou Medical College, Hangzhou 310000, China.; ^4^Department of Neurosurgery, Xiangya Hospital, Central South University, Changsha, Hunan 410000, China.; ^5^Department of Scientific Research, Xiangya Hospital, Central South University, Changsha, Hunan 410000, China.; ^6^National Clinical Research Center for Geriatric Disorders, Xiangya Hospital, Central South University, Changsha, Hunan 410000, China

**Keywords:** melatonin, cartilage, cartilage homeostasis, inflammation, cartilage-related genes, human mesenchymal stromal cells

## Abstract

Cartilage is a relatively simple connective tissue that plays a variety of roles in the human body, including joint support and protection, load bearing of the intervertebral discs, joint lubrication, formation of the external structure of the ears and nose and support of the trachea. The maintenance of cartilage homeostasis is therefore crucial. Cartilage-related diseases are difficult to diagnose and treat because their molecular and pathological mechanisms are not fully understood. Melatonin, which has a wide range of physiological effects, is an endocrine hormone mainly secreted by the pineal gland. Its biological effects include its antioxidant, antiaging, analgesic, and hypnotic effects and its ability to stabilize the circadian rhythm. In recent years, research on cartilage homeostasis and melatonin has been increasing, and melatonin has gradually been used in the treatment of cartilage-related diseases. Therefore, this article will briefly review the role of melatonin in cartilage homeostasis, including its anti-inflammatory effects and effects in protecting cartilage from damage by other factors and promoting chondrocyte growth and the expression of cartilage-related genes. Based on the above, the current status and future developmental direction of melatonin in the treatment of cartilage-related diseases are also discussed, demonstrating the broad prospects of melatonin in maintaining cartilage homeostasis and treating cartilage injury-related diseases.

Cartilage, which is composed of chondrocytes, cell stroma and a gel-like matrix, is a type of connective tissue with support functions. Cartilage does not contain blood vessels, lymphatic vessels or nerves and is generally classified according to its cell stroma as articular cartilage, hyaline cartilage or elastic cartilage [1, 2]. Hyaline cartilage is clinically regenerated from the surrounding perichondrium, suggesting the presence of stem/progenitor cells in the perichondrium [3, 4].

Articular cartilage is mainly present in joints and ligaments. It has a robust physiological structure and mechanical properties, which allow flexibility in its movement. Articular cartilage supports and protects joints but has a very poor self-repair ability after injury or disease [5, 6]. Hyaline cartilage is covered by a layer of dense connective tissue and serves mainly as a temporary scaffold in the embryonic period, after which it is replaced by bone [7]. It is semitransparent in the human body, easily damaged and brittle. Hyaline cartilage is widely distributed in joints, costal cartilage and the adult respiratory tract. Elastic cartilage is found in the auricle and epiglottis, and a large number of elastic fibers are interwoven into its stroma.

Cartilage damage can be induced and repaired by human mesenchymal stromal cells (MSCs), but cartilage exhibits limited proliferation throughout the aging process [8, 9]. The discovery of induced pluripotent stem cells (iPSCs) in 2006, however, has provided many new approaches for cartilage tissue engineering therapy. The use of iPSCs has helped overcome previous limitations and shows potential for clinical application [10]. The replacement of cartilage with other types of cartilage—such as the reconstruction of elastic cartilage by transplantation of chondrocytes derived from cultured hyaline cartilage—can also be completed under the appropriate conditions [11]. The degradation of cartilage in the human body is affected by aging, genetic susceptibility, daily activities and so on. Various health problems caused by cartilage-related diseases and their large burden in terms of social and medical costs have elicited widespread concern [12].

Melatonin is a ubiquitous molecule in nature found in almost all living organisms. It is an indolamine present in all systems of an organism due to its amphiphilic characteristics and diffusion. It can also be produced by chondrocytes and many other tissues and organs. Cartilage cells produce melatonin in response to circulating exogenous melatonin and can upregulate melatonin receptor expression. Melatonin regulates cartilage growth and maturation through melatonin receptor 1 (MTNR1A) and melatonin receptor 2 (MTNR1B) [13]. MTNR1A plays an important role in controlling the circadian rhythm, and MTNR1B is closely related to the cyclic activity of melatonin in the body [14]. As melatonin secretion shows diurnal changes, the proliferation and growth of chondrocytes also follow day and night patterns. Calcium and phosphate mineralization rates are also increased in the dark phase of the light and dark cycle [15]. The circadian expression of melatonin can regulate various key biological processes, including inflammation, and circadian rhythm disorders are closely related to the etiology of inflammatory arthritis [16]. The hormone melatonin is secreted by the pineal gland under control of the circadian rhythm; melatonin is released at night and suppressed during the day. Evidence indicates that melatonin levels gradually decrease with age [17]. Furthermore, the effects of melatonin differ between cartilage types [13].

This article reviews the basic structure and function of melatonin and its role in cartilage homeostasis.

## Basic structure and function of melatonin

Melatonin, first discovered in the 1950s, is a ubiquitous molecule in nature [18]. The chemical name and molecular formula of melatonin are N-acetyl-5-methoxytryptamine and C_13_H_16_N_2_O_2_, respectively. Its properties include a relative molecular mass of 232.27 grams per mole and a melting point of 116-118, and pure melatonin consists of pale yellow leaf-like crystals [19].

Melatonin is concentrated in the pineal gland of vertebrates (especially mammals) but is also locally synthesized in other cells and tissues [20]. It is mainly converted from tryptophan in pineal gland cells through complex biochemical reactions. In some cases, melatonin can also be synthesized in the guts and lungs [21], and the latest research shows that reprogramming of the gut microbiota also affects the level of melatonin in the gut [22]. Melatonin synthesis by the pineal gland is controlled by the suprachiasmatic nucleus and occurs at night through synchronization of the optic nerve and hypothalamus bundle and the light and dark cycle. This unique characteristic allows adaptation of the physiological functions of melatonin depending on daily and seasonal needs [23, 24]. The acute inflammatory response drives the transcription factor nuclear factor κ-light-chain-enhancer of activated B cells (NF-κB), which switches melatonin synthesis from pinealocytes to macrophages/microglia and, upon acute inflammatory resolution, back to pinealocytes [25]. This bidirectional communication between the pineal gland and the immune system is termed the immune-pineal axis. Studies have shown that bone marrow is another component of the immune-pineal axis, in which pineal melatonin may have a role in surveillance [26, 27]. However, further investigation regarding the role of melatonin in hematopoiesis is required. Furthermore, over the course of the inflammatory response, TNF inhibits nocturnal pineal synthesis and induces the synthesis of melatonin by macrophages and other immunocompetent cells [25, 28]. In mammals, nuclear translocation of NF-κB blocks noradrenaline-induced melatonin synthesis in pinealocytes, which induces melatonin synthesis in macrophages [29]. In addition, melatonin reduces NF-κB activation in pinealocytes and immune competent cells. In mammals, the major site of melatonin metabolism is the liver. Melatonin metabolism is carried out through complex pathways in the cell cytoplasm, endoplasmic reticulum and mitochondria but can also take place at the production site or in the skin [30].

In recent years, research on melatonin has been increasing. Melatonin has been shown to have a wide range of effects on biological rhythm, reproduction, immunity, digestion, central nervous system function and antioxidant and antitumor activities [31]. In addition, melatonin has been reported to be involved in the regeneration of various tissues in the nervous system, liver, bone, kidney, bladder, skin and muscle [32].

## Mechanism of action of melatonin

Melatonin exerts its physiological effects in a variety of ways. It acts at melatonin membrane receptors (MTNR1A and MTNR1B), melatonin nuclear binding sites (e.g., retinoid Z receptor [RZR] and retinoid acid receptor-related orphan receptor [ROR]) and in non-receptor-dependent pathways. The best characterized pathway is the activation of two types of membrane-specific receptors: the high-affinity MTNR1A receptor and low-affinity MTNR1B receptor [33, 34].

MTNR1A, which is encoded in human chromosome 4 (4q35.1) and consists of 351 amino acids, is widely distributed and found in the tubercle of the anterior pituitary, suprachiasmatic nucleus of the hypothalamus (the anatomical site of the circadian rhythm), cortex, thalamus, substantia nigra, amygdala, hippocampus, cerebellum, cornea and retina [35]. Activation of MTNR1A can inhibit the discharge of suprachiasmatic nucleus neurons, thus inhibiting hormone secretion and promoting cardiovascular stimulation [36, 37].

MTNR1B is encoded in human chromosome 11 (11q21-q22) and consists of 363 amino acids. The Mel1b receptor is mainly distributed in the retina, followed by the hippocampus, cortex, paraventricular nucleus and cerebellum [38]. Activation of MTNR1B can regulate the circadian rhythm, relax coronary artery vessels, induce proliferation of spleen cells and inhibit the release of retinal dopamine [39]. Although an MTNR1C-binding partner has been discovered and identified as quinone reductase 2 in several species, but not humans, its function and mechanism of action remain unclear [40].

Melatonin exerts its main effects, some of which are dependent on the retinoid-related orphan nuclear hormone receptor family, by binding MTNR1A and MTNR1B [41, 42]. However, recent studies have proven that RORα is not a melatonin receptor [43]. Melatonin exerts its free-radical scavenging and antioxidant effects through receptor-independent pathways. Melatonin and its metabolites are powerful free radical scavengers and indirect antioxidants [44]. Melatonin very effectively reduced oxidative stress in all experimental and clinical settings in which it has been tested and has an advantage over other antioxidants, since not only melatonin but also some of its metabolites are scavengers of toxic species [45]. In a rat model exposed to polluted air, the enrichment score for antioxidant genes obtained from lung gene expression data (GTEx) was significantly correlated with the levels of MTNR1A but not MTNR1B [46].

## Cartilage homeostasis

As noted previously, cartilage is composed of chondrocytes and a gel-like matrix. The main components of the cartilage matrix are collagen and proteoglycan. Collagen type II (COL2A1) is the most abundant component of the cartilage matrix, in which it forms a fibrous network structure. Proteoglycans attract a large number of water molecules to form a gel, maintaining the swelling ability and elasticity of cartilage [47, 48]. The cartilage matrix provides a home for chondrocytes, and its stability is directly related to the metabolic balance of chondrocytes [49]. Chondrocyte survival and metabolic equilibrium in the cartilage matrix directly affect the occurrence and development of cartilage-related diseases. The main factors currently known to affect cartilage homeostasis are degenerative disease and inflammation.

Cartilage degeneration is characterized by superficial cartilage defects or fibrosis at the initial stage. This is followed by the extension of fissures to subchondral bone and ulceration. The cartilage gradually becomes thinner, eventually leading to full-thickness cartilage defects and denudation as disease progresses [50, 51]. Cartilage degeneration is an important early change in many osteoarticular diseases, such as osteoarthritis (OA). In the early stage of OA, cartilage covered by a meniscus easily degenerates. Collagen fibers degenerate and gradually ulcerate [52]. As the disease progresses, the following processes occur: 1) the collagen fiber structure is damaged, 2) proteoglycan is excessively degraded, 3) the mechanical properties of cartilage are compromised, 4) the subchondral bone erodes upward and forms a medullary cavity-like structure, 5) articular cartilage is gradually vascularized, 6) cartilage is damaged and thinned, 7) the subchondral bone is gradually exposed, 8) the joint space is narrowed, and 9) osteophytes are formed [53]. The degradation and destruction of cartilage contribute to an inflammatory environment in joints, leading to hyperplasia and angiogenesis of the joint synovium. Inflammatory factors produced in pathological processes can directly act on chondrocytes, causing imbalance between anabolism and catabolism, further aggravating degradation and destruction of the cartilage extracellular matrix (ECM) [54].

Inflammatory factors have a highly disruptive effect on the steady-state environment of cartilage. Under conditions of trauma, cartilage degeneration, and hyperosteogeny, the synovial membrane produces an inflammatory reaction after its stimulation. Synovial cells secrete a large amount of cytokines, chemokines, reactive oxygen species and matrix metalloproteinases (MMPs) into the synovial fluid [55, 56]. Levels of inflammatory factors such as interleukin (IL)-1, IL-6 and tumor necrosis factor-α (TNF-α) increase, promoting the expression of collagenase and proteoglycan enzymes (e.g., depolymerizing protein-like metalloprotease [ADAMTS], MMP-13, MMP-9, and MMP-3) in chondrocytes. This results in the degradation of COL2A1 and proteoglycans, cartilage matrix imbalance and changes in the cartilage structure [57-59]. With the aggravation of ongoing disease, cartilage is further eroded, the collagen fiber network structure is destroyed, inflammatory factor secretion continues to increase, and cartilage homeostasis is further disrupted [60]. TNF-α and IL-1β can promote chondrocytes to release more inflammatory factors, such as nitric oxide (NO), MMPs and ADAMTS. IL-1β can also promote the secretion of TNF-α, which itself exerts effects on IL-1β, promotes the synthesis of MMPs, inhibits the synthesis of proteoglycans and causes cartilage loss [61, 62].

## Effect of melatonin on cartilage homeostasis

### Anti-inflammatory effect of melatonin

Melatonin has pro- and anti-inflammatory effects depending on its dosage and the cell status. Melatonin can promote the early stage of inflammation. It is quite beneficial in reducing inflammation and preventing the complications of chronic inflammation [63]. In terms of cartilage-related inflammation, the role of melatonin in rheumatoid arthritis (RA) is still controversial. Some studies have shown that melatonin plays an inflammatory role in RA, promoting disease progression [63, 64], while other studies have demonstrated that melatonin plays an anti-inflammatory role in RA [65, 66]. A number of studies have also confirmed that melatonin plays an anti-inflammatory role in OA and other arthritides [67-69].

OA, one of the most common joint diseases, is very common in elderly individuals and one of the most ordinary causes of disability in this population. The main pathological changes in OA are a reduction in articular chondrocytes and cartilage matrix degradation [70]. Inflammatory mediators produced by chondrocytes play a key role in the development of OA. IL-1β and TNF-α are the two most effective catabolic factors, but IL-1β has a stronger inhibitory effect on cartilage formation than TNF-α [71]. Studies have shown that melatonin may play a protective role against OA through its regulatory effect on oxidative stress, the reduced secretion of proinflammatory cytokines and alleviation of mitochondrial dysfunction [58]. Melatonin may inhibit the activation of MMPs by reducing the accumulation of ROS and increasing the expression of superoxide dismutase, thus avoiding the excessive degradation of ECM [72].

Sirtuin 1 (SIRT1) is a nicotinamide adenine dinucleotide (NAD^+^)-dependent histone deacetylase in the peripheral tissues that controls many physiological pathways, including circadian rhythms [73]. Melatonin can also be expressed by SIRT1-dependent nicotinamide phosphoribosyltransferase (NAMPT) and nuclear factor of activated T cells 5 (NFAT5). Signal transduction reduces the production of MMPs (MMP-1, MMP-2, MMP-3, MMP-9, MMP-13) induced by IL-1β, thus preventing the occurrence and development of OA [74]. Guo et al.[75] proved that melatonin regulates the expression and activity of SIRT1 by inhibiting the NAMPT and NFAT5 signaling pathways in chondrocytes, effectively reducing the IL-1β-induced production of MMP-3 and MMP-13 in cartilage. This suggests that melatonin has a protective effect in the cartilage of OA patients. MMPs promote cartilage matrix degradation and articular cartilage degeneration in OA patients [76]. Excessive secretion of MMPs leads to the degradation of ECM, and the fragments produced by this degradation have an important impact on the normal metabolism of chondrocytes. Loss of the normal environment in which chondrocytes depend for survival leads to a decrease in their number, resulting in pathological and biomechanical changes, such as thinning of the cartilage layer, the generation of fissures and changes in the distribution and arrangement of chondrocytes (e.g., clustering, reduction in number).

**Figure 1.**
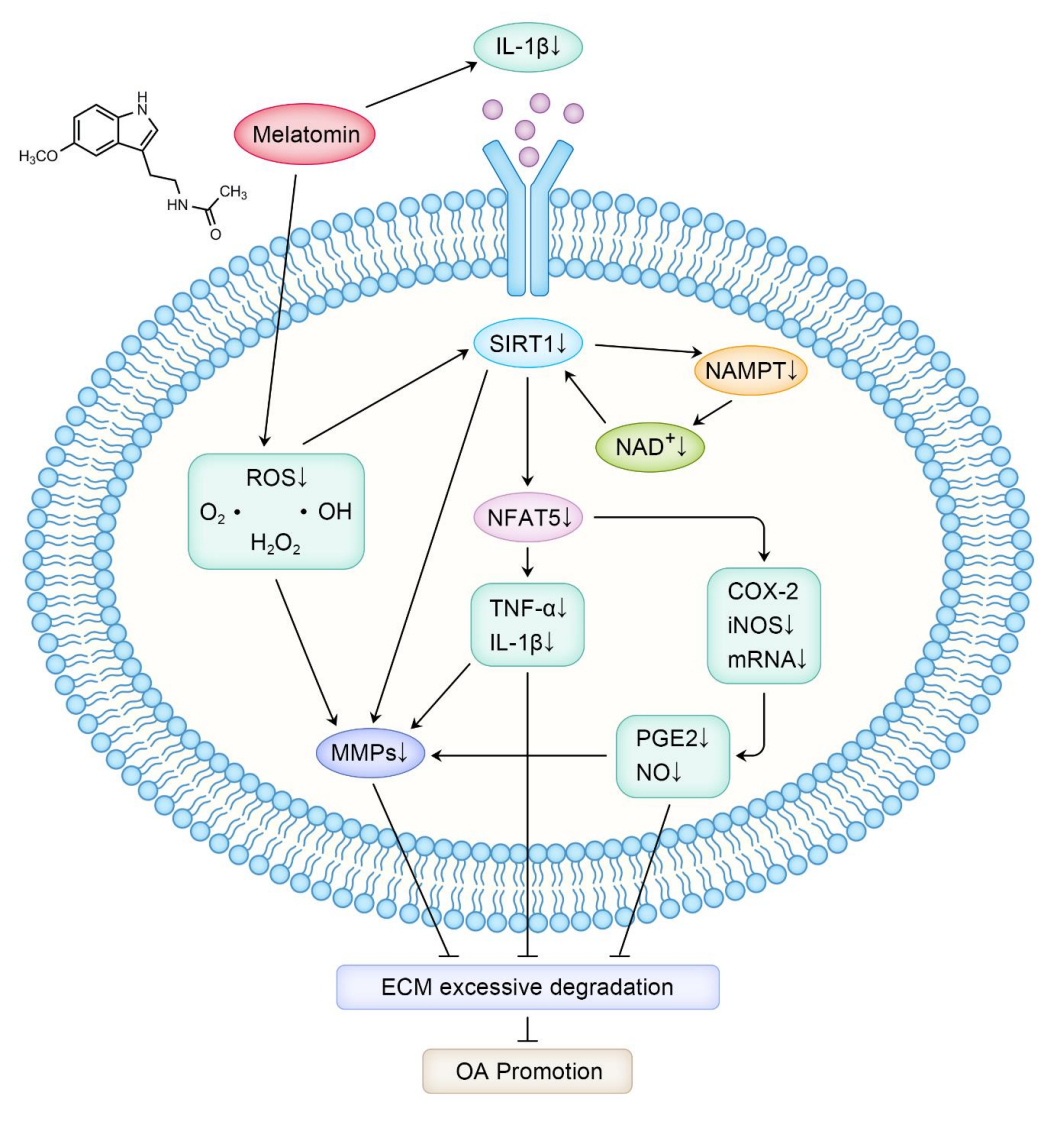
The melatonin signaling pathway in the occurrence and development of OA.

Lim et al. [77] proposed that melatonin plays a protective anti-inflammatory role through the SIRT1 pathway, as evidenced in oxidative stress-stimulated chondrocytes and rabbit OA models. Melatonin significantly inhibits the cytotoxicity of hydrogen peroxide (H_2_O_2_) and proteins and messenger ribonucleic acid (mRNA) expression of inducible nitric oxide synthase (iNOS) and cyclooxygenase-2 (COX-2). It has also been found to inhibit the production of NO and prostaglandin E2 (PGE2), downstream products of iNOS and COX-2. Intraarticular injection of melatonin significantly reduced cartilage destruction in rabbit OA models, while sirtinol and SIRT1 small interfering RNA (siRNA) reversed the effect of melatonin. Inflammation and oxidative stress often interact and synergistically determine the occurrence and development of aging-related chronic diseases. The use of potent anti-inflammatory and antioxidant functional compound drugs with high bioavailability, such as melatonin, may become a promising, safe and effective intervention strategy to delay aging and the occurrence and development of OA. The specific signaling pathway of melatonin in the pathogenesis of OA is shown in Figure 1.

### Role in protecting cartilage from other factors

In addition to protecting cartilage from the anti-inflammatory effects of OA and arthritides other than RA, melatonin protects cartilage from other sources of damage. Intraarticular injection of glucocorticoids (GCs) may relieve pain and inflammation in patients with OA, but the long-term use of GCs might inhibit the synthesis of major cartilage matrix components in a dose-dependent manner. Melatonin was found to exert a protective effect against GC-induced chondrocyte matrix degeneration [78, 79]. An in vitro experiment discovered that melatonin pretreatment could effectively reduce hormone-induced cartilage matrix loss. The results showed that dexamethasone treatment reduced the proteoglycan and COL2A1 content in the cartilage matrix of mice, but melatonin pretreatment reversed the inhibitory effect of dexamethasone [80]. Although some progress has been made, the antidegradation effect of melatonin on the extracellular matrix is worthy of further study. For instance, whether melatonin still has a protective effect on articular cartilage in the face of OA induced by aging and trauma is unclear. Additionally, determining whether melatonin can better protect against the loss of cartilage matrix when combined with other drugs also needs further study.

Due to its antioxidant properties, melatonin can also protect chondrocytes from toxicity. Studies have shown that melatonin alleviates IL-1β- and H_2_O_2_-induced cytotoxicity in a dose-dependent manner [74, 77]. Melatonin pretreatment significantly reduced H_2_O_2_-induced MSC apoptosis in a dose-dependent manner and effectively inhibited H_2_O_2_-induced ROS production, the Bax/Bcl-2 expression ratio, caspase-3 activation and phosphorylated P38MAPK expression in MSCs [81]. In cartilage endplate cells (EPCs) under oxidative stress, melatonin therapy was capable of reducing the incidence of apoptosis and inhibiting EPC calcification through SIRT1-mediated autophagy, indicating the protective effect of melatonin against EPC apoptosis and calcification [82]. Melatonin (1 nM) could also prevent cartilage degeneration and correct phenotypic abnormalities in chondrocytes, but long-term use of melatonin or the use of large doses (1 mM) of melatonin led to serious subchondral bone erosion [83]. Therefore, the dose and action time of melatonin must be strictly controlled depending on the disease, patient, administration method and so on. Only once these measures have been taken can further aggravation of the diseases attributed to melatonin be effectively avoided.

### Role in promoting chondrocyte growth and the expression of cartilage-related genes

Melatonin plays an important role in promoting the growth and development of cartilage. A chick embryo animal model revealed that the melatonin concentration and chick embryo cartilage development were increased in dark environments compared to light environments [84]. During the differentiation of MSCs, melatonin promoted cartilage differentiation and is thus suitable for therapeutic applications in cartilage regeneration. Under conditions of normal cartilage differentiation, quantitative analysis of glycosaminoglycans (GAGs) and real-time polymerase chain reaction analysis proved that melatonin treatment significantly increased the expression of genes involved in cartilage formation and differentiation. These genes included aggrecan (ACAN), COL2A1, collagen type X (COL10A1), sex-determining region Y (SRY)-box 9 (SOX9), runt-related transcription factor and bone morphogenetic protein 2, a potent inducer of chondrogenic differentiation. Moreover, the melatonin group exhibited more induced cartilage tissue that was enriched in GAG, COL2A1 and COL10A1 expression [85]. Pei et al.[86] discovered that melatonin could not only upregulate cartilage differentiation but also inhibit osteogenic differentiation. In addition, they observed upregulation of transforming growth factor β1 expression in melatonin-treated cells. Therefore, they suggested that melatonin promotes matrix synthesis by articular chondrocytes through the transforming growth factor β signaling pathway. Thus, we believe that melatonin is an important regulator of MSC differentiation with potential application value in promoting bone formation and fracture healing.

Studies have demonstrated that melatonin can successfully restore the inhibitory effects of IL-1β and TNF-α on MSC activity, promote the mRNA expression of chondrocyte-specific marker genes in MSCs, facilitate the differentiation of MSCs into chondrocytes, inhibit the expression of MMPs, and maintain the activity of superoxide dismutase [87]. Liu et al.[88] proved that melatonin can 1) promote the expression of cartilage matrix and cartilage formation-related genes in MSCs, 2) downregulate the mRNA expression of MMP-1, MMP-2, MMP-9 and MMP-13, 3) reduce the accumulation of reactive oxygen species, 4) promote the expression of superoxide dismutase, and 5) protect chondrocytes in medium, promoting the differentiation of MSCs into chondrocytes. In the inflammatory environment induced by IL-1β, melatonin can not only promote the synthesis and accumulation of cartilage matrix but also 1) upregulate expression of the marker of cartilage formation COL2A1 at the mRNA and protein levels in the presence of IL-1β; 2) regulate the expression levels of the other markers of cartilage formation markers ACAN, SOX9 and COL10A1; and 3) inhibit the apoptosis of MSCs induced by IL-1β during the process of whole-cartilage formation [67]. In conclusion, with its strong inhibitory effect on MMPs, melatonin may be a promising therapeutic strategy to protect proteins from enzyme proteolysis. At the gene level, melatonin promotes cartilage formation and the differentiation of human MSCs by upregulating miR-526b-3p and miR-590-5p. Consistent with this is the finding that miR-526b-3p or miR-590-5p inhibitor could almost eliminate the positive effect of melatonin in promoting cartilage formation and the differentiation of human MSCs [89]. Future research could focus on exploring the translational value of these miRNAs, which are expected to be used as diagnostic markers and therapeutic targets for cartilage regeneration in various cartilage defect diseases. High concentrations of melatonin (4×10^-4^ M), however, can inhibit chondrocyte proliferation and the mRNA expression of COL2A1, ACAN, and SOX9 [90]. Excessive melatonin not only leads to serous subchondral bone degeneration at the cellular level but also inhibits the expression of chondrocyte-related genes at the molecular level. These findings will further ensure the precise use of melatonin at a therapeutic dose.

## Targeting melatonin in cartilage-related diseases

The key functions of melatonin, such as its reduction of inflammation and apoptosis and maintenance of metabolic balance, make it a potential treatment for OA [67, 69, 77]. Studies have discovered that melatonin can maintain the survival of MSCs and promote their osteogenic differentiation under the inflammatory conditions induced by IL-1β. Melatonin may become a new drug for OA treatment and bone tissue regeneration [91]. As exercise helps protect against articular cartilage degeneration in animals and humans [92], melatonin therapy and exercise have preventive and synergistic effects against cartilage degeneration in OA [83, 93]. Melatonin also plays an important role in cartilage injury-related diseases due to its function in promoting cartilage cell growth and the formation and differentiation of MSCs into cartilage. In a rat model of acute rotator cuff tear, melatonin-loaded aligned polycaprolactone electrospun fiber membranes began to inhibit macrophage infiltration at the tendon-bone interface early in healing, leading to the increased formation of cartilage regions, maturation of collagen and improved biomechanical strength of regenerated skeletons [94]. Since melatonin promotes cartilage formation and the differentiation of MSCs, its application may provide a new approach for rotator cuff repair in plastic surgery. At present, hydrogels or hydrogel/microparticle systems containing melatonin have become suitable substitute materials for cartilage in tissue engineering due to their noncytotoxicity, sustained drug release capability and high biological activity [95]. A hyaluronic acid hydrogel system containing various amounts of polylatide-co-glycolide micro/nanoparticle-coated with chitosan-acrylic acid increased GAG synthesis, promoted cartilage cell growth and proliferation, and improved the mechanical properties of cartilage tissue [96]. In a study of heterogeneous MSC transplantation, Pescador et al.[97] concluded that the use of xenogeneic MSCs embedded in an elastin-like recombinamer-based hydrogel led to the successful regeneration of hyaline cartilage in osteochondral lesions. Novel therapeutic tools for osteochondral regeneration have arisen from the combination of MSCs and highly specialized smart biomaterials, such as hydrogel-forming elastin-like recombinamers (ELRs), which can serve as cell carriers. Recently, hydrogels and hydrogel/microparticle systems have been highly recommended as alternatives for cartilage tissue engineering in tissue engineering and regenerative medicine. The functions of melatonin in cartilage-related diseases are shown in Table 1.

**Table 1:** The functions of melatonin in cartilage-related diseases.

Cartilage-related disease	Function of melatonin	References
OA	Reducing inflammation, maintaining metabolic balance and reducing apoptosis	[67, 69, 77]
	Maintaining the survival of MSCs and promoting their osteogenic differentiation	[91]
Rotator cuff repair	Inhibiting macrophage infiltration at the tendon-bone interface and increasing the formation of cartilage regions	[94]
Cartilage tissue engineering	Hydrogels or hydrogel/microparticle systems, cartilage regeneration	[95,96,97]

## Conclusions

The incidence of cartilage injury increases with aging. Due to the lack of blood vessels, the self-repair capability of cartilage after injury is quite ineffective. Once unbalanced, cartilage homeostasis is hard to restore; thus far, there are no satisfactory drugs or therapeutic methods for the treatment of cartilage-related diseases such as OA. Melatonin, which possesses biological effects including its antioxidant, antiaging, analgesic, and hypnotic effects and the ability to stabilize the circadian rhythm, shows unique prospects in maintaining cartilage homeostasis. Melatonin plays an important role in protecting chondrocyte growth and promoting the expression of cartilage-related genes and regeneration of cartilage, which are closely related to its anti-inflammatory and antioxidative stress effects. Thus, melatonin is expected to become a promising, safe and effective intervention strategy to delay aging and the occurrence and development of cartilage-related diseases. Although some progress has been made, many mechanisms underlying its effects remain unclear, and more scientific research and clinical trials are needed to explore the specific role of melatonin in cartilage homeostasis and cartilage injury-related diseases.
